# Meta-analysis of transcriptome datasets: An alternative method to study IL-6 regulation in coronavirus disease 2019

**DOI:** 10.1016/j.csbj.2020.12.010

**Published:** 2020-12-24

**Authors:** Hui Liu, Shujin Lin, Xiulan Ao, Xiangwen Gong, Chunyun Liu, Dechang Xu, Yumei Huang, Zhiqiang Liu, Bixing Zhao, Xiaolong Liu, Xiao Han, Hanhui Ye

**Affiliations:** a Ganzhou Fifth People’s Hospital, China; b Mengchao Hepatobiliary Hospital of Fujian Medical University, China; c College of Biological Science and Engineering, Fuzhou University, China

**Keywords:** IL-6, Meta-analysis, Transcriptome, Respiratory disease, Cardiovasculardisease, Resveratrol

## Abstract

In coronavirus disease 2019 (COVID-19) patients, interleukin (IL)-6 is one of the leading factors causing death through cytokine release syndrome. Hence, identification of IL-6 downstream from clinical patients’ transcriptome is very valid for analyses of its mechanism. However, clinical study is conditional and time consuming to collect optional size of samples, as patients have the clinical heterogeneity. A possible solution is to deeply mine the relative existing data. Several transcriptome-based studies on other diseases or treatments have revealed different genes to be regulated by IL-6. Through our meta-analysis of these transcriptome datasets, 352 genes were suggested to be regulated by IL-6 in different biological conditions, some of which were related to virus infection and cardiovascular disease. Among them, 232 genes were not identified by current transcriptome studies from clinical research. *ICAM1* and *PFKFB3* were the most significantly upregulated genes in our meta-analysis and could be employed as biomarkers in patients with severe COVID-19. In general, a meta-analysis of transcriptome datasets could be an alternative way to analyze the immune response and complications of patients suffering from severe COVID-19 and other emergency diseases.

## Graphical abstract

**Figure:**
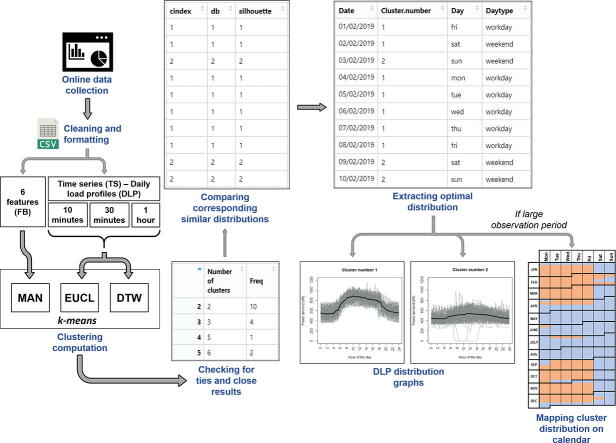

## 1 Introduction

Coronavirus disease 2019 (COVID-19) is wreaking havoc in healthcare and economic systems worldwide [1]. Analyses of the mode of action of the pathogen that causes COVID-19, severe acute respiratory syndrome coronavirus 2 (SARS-CoV-2), are crucial to develop therapeutics.

Studies have shown that high ratio of COVID-19 patients develop severe disease and a proportion of such patients die [1], [2]. Cytokine release syndrome (CRS; also known as “cytokine storm”) can be triggered by various factors, and occurs when large numbers of white blood cells are activated and release inflammatory cytokines which, in turn, activate yet more white blood cells [3]. CRS has been suggested to be one of the principal causes for severe COVID-19, which leads to increased risks in patients with cardiovascular disease and diabetes mellitus.

Clinical evidence has revealed interleukin (IL)-6, a major chemokine in CRS, to be a critical biomarker and predictor for severe COVID-19 [4], [5], [6], [7]. IL-6 is pleiotropic, and a vital pro-inflammatory factor that regulates hematopoiesis, respiration, as well as the response to infection and cancer [8], [9], [10], [11]. Hence, studying the key regulatory genes downstream of IL-6 is important for analyses of its mechanism of action and the design of drug targets against SARS-CoV-2.

Analysis of the transcriptome is a high-throughput, molecular-biological approach to deconstruct the intricate gene-regulation network. So far, there are studies applied this method to resolve this problem and identified several differential expressed genes in COIVID-19 patients [12], [13], [14], [15], [16]. However, to study severe COVID-19 in this way, a series of complex problems must be taken into consideration to save time and ensure accuracy, such as the heterogeneity of clinical samples and the difficulty of obtaining appropriate samples. IL-6 has been shown to be a key biomarker for severe COVID-19, so there is an alternative method to integrate the common factors regulated by IL-6 from other diseases or treatment studies.

Several transcriptome datasets related to IL-6 regulation are available. Study used Illumina HT12.0 microarrays to ascertain the differentially expressed genes (DEGs) in dendritic cells upon treatment with tumor necrosis factor (TNF)-α, IL-1, IL-6, prostaglandin E2 or interferon (IFN)-γ [17]. Researcher investigated the transcriptional changes of renal tubular epithelial cells stimulated by the proinflammatory cytokines IL-6, IFN-γ or TNF-α. They showed that nuclear factor-kappa B (NF-κB), signal transducer and activator of transcription (STAT)1 and STAT3 were the key genes influencing gene expression during renal aging [18]. A similar study on dendritic cells used transcriptome analyses to validate regulation of STAT3 expression by IL-6 [19]. Study used an antibody against the IL-6 receptor to block the IL-6 pathway and investigated the transcriptome in different tumor cells. They found that the STAT3 signaling pathway was highly active in most cell lines, suggesting that IL-6 regulated signaling [20]. Researchers investigated IL-6 regulation by transcriptome analyses on apoptosis of INA6 cells through the p53/STAT3 pathway [21]. Human monocyte-derived macrophages have also been studied under IL-6 stimulation by genome-wide transcriptome analyses [22]. The transcriptome research mentioned above suggests that IL-6 regulation varies under different conditions. So far, scholars have not integrated those results to find the common genes involved in IL-6 regulation to support relevant application in COVID-19.

Meta-analyses are popular methods to statistically integrate results considering the sample size in each experiment. It has enabled considerable progress to be made in identifying and replicating common genetic variants associated with susceptibility to certain diseases. Researchers undertook a meta-analysis to study infection by the West Nile virus by comparing new listed genes between samples from infected patients and those from healthy controls [23]. A meta-analysis can also be very helpful for studying functional annotation among multi-gene expression. Researchers employed a meta-analysis to study liver and heart tissues by collecting transcriptome data [24]. Researchers utilized an integrative meta-analysis of expression data to identify the various genes expressed [25]. However, they just average the fold change or statistical possibility from multiple experiments without standard processes of meta-analysis.

A standard meta-analysis is a statistical procedure for combining datasets from multiple studies considering the sample size. If the treatment effect (or effect size) is consistent from one study to another study, a meta-analysis can be used to identify this common effect. Due to the differences in the genetic background of human samples, a meta-analysis is required.

We undertook a standard meta-analysis by collecting transcriptome datasets from IL to 6-treatment experiments. We discovered that this method can help to identify IL-6-regulated genes in COVID-19.

## 2 Materials and methods

### 2.1 Sample collection and ethical approval

Blood samples from patients with severe COVID-19 admitted to Ganzhou Fifth People’s Hospital (Chizhu, China) from January 2020 were used for analyses. The diagnostic criteria for the selected cases were based on the *COVID-19 Treatment Guide* (4th Edition, 2020). Ethical approval of this study was obtained from the Ganzhou Fifth People’s Hospital Ethics Committee.

### 2.2 Collection of microarray data

The keyword we used for searching was “IL-6”, and we limited the research type to “expression profiling by array”, in the Gene Expression Omnibus (GEO) database from the National Center for Biotechnology Information (www.ncbi.nlm.gov/geo/). the results provide associated with IL-6 treatment genome-wide expression data are 13 sets of chips.

The inclusion criteria for the dataset were: (i) the dataset must be genome-wide mRNA-expression chip data supported by the literature; (ii) the original or standardized dataset must be considered; (iii) each dataset must include > 3 samples.

After these searches, we obtained the gene-expression microarray data from the GEO database. Simultaneously, we established a corresponding control group, from which four independent microarray datasets with raw data were selected (Table 1).

**Table 1:** Summary of transcriptome datasets with IL-6 treatment used in this study. The numbers of experiment and control indicate the replicates in each dataset. All treatment used exogenous IL-6 except the * and # marked one. *, IL-6 receptor antibody; #, IL-6 level determined in patients. (-1/2/3 in the same Datasets were different treatment experiments).

Datasets	Experiment	Control	Sample	Treatment
GSE76340	3	3	Immature dendritic cells	TNF-α, IL-1, IL-6, prostaglandin E2 and IFN-γ
GSE68942-1	3	3	HK-2	IFN/IL6
GSE68942-2	3	3	HK-2	IFN/TNF/IL6
GSE68941	3	3	HK-2	TNF/IL6
GSE68940	3	3	HK-2	IL6
GSE62941-1	3	3	KPL4	Medi5117
GSE62941-2	3	3	NCI-H1650	Medi5117
GSE62941-3	3	3	DU145	Medi5117
GSE29793	3	3	INA6	withdraw IL6
GSE45466	8	8	Monocyte-derived dendritic cell	IL6
GSE12385	18	18	PMBC	IL6 level
GSE10685	3	3	Human skeletal muscle	IL6
GSE8515	5	5	macrophage	IL6

COVID-19 related Transcriptome Datasets were download from public website including GSE147507, E-MTAB-8871, CRA002390 and HRA000143.

### 2.3 Meta-analysis of differential gene expression

R language (R Center for Statistical Computing, Vienna, Austria) was used to process the data, conduct statistical analyses, and obtain the path through which the data changed together. A meta-analysis was conducted on the results with the random-effects model to obtain the combined differential expression of genes, statistical tests, and to input the genes into the Database for Annotation, Visualization and Integrated Discovery (DAVID) website to obtain the possible enriched pathway.

### 2.4 Gene enrichment using the gene ontology (GO) and kyoto encyclopedia of genes and genomes (KEGG) databases and disease analysis

In the present study, DEGs were defined through functional interpretation using DAVID (http://david.abcc.ncifcrf.gov/) [26].We carried out statistical analyses, with p < 0.05 denoting significance. We obtained a gene-symbol list that was uploaded into DAVID. We also used the GO and KEGG databases. To unify their format, a functional annotation chart was used for uploading and analyses. Interactions between disease and chemicals were analyzed using the Comparative Toxicogenomics Database (CTD) (ctd.mdibl.org/). The co-expression network were constructed by STRING database (https://string-db.org/cgi/)

### 2.5 RNA extraction and quantitative reverse transcription-polymerase chain reaction (qRT-PCR)

An RNA Isolation kit was applied to extract total RNA according to manufacturer instructions. A NanoDrop™ ND-1000 spectrophotometer (Thermo Fisher Scientific, Wilmington, MA, USA) was used to determine the quantity and purity of RNA. Expression of complimentary DNA was measured using a Prime Script™ RT kit (TaKaRa Biotechnology, Otsu, Japan). qPCR was undertaken on a 7900HT fast RT qPCR instrument (Applied Biosystems, Foster City, CA, USA) according to manufacturer instructions. The RNA of actin was used as an internal loading control.

## 3 Results

### 3.1 Less overlapping in different COVID-19 transcriptome studies

**Fig. 1:**
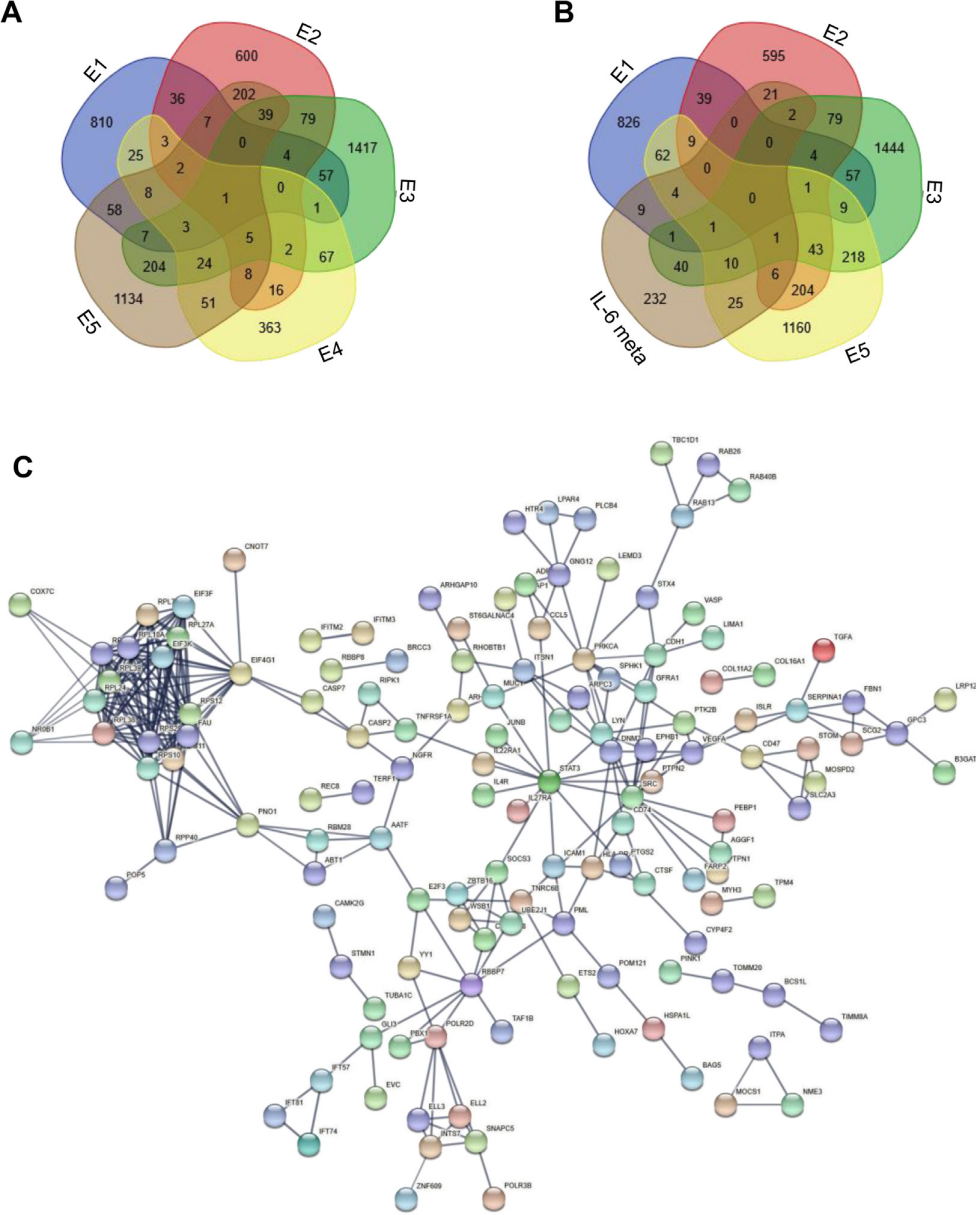
DEGs in clinical COVID studies and meta-analysis. (A) Venn Diagram of DEGs from five clinical studies. (B) Venn Diagram of DEGs from meta-analysis and five clinical studies. (C) co-expression network of DEGs.

We collected the transcriptional profiles from five COVID-19 clinical studies which used samples including peripheral blood, PBMC and ronchoalveolar lavage fluid. In totally, <20 COVID-19 patients were enrolled to be studied. Fig. 1A showed that only one gene TNFSF10 is differential expressed genes (DEG) in all five studies. Ten DEG were identified in four studies, the most DEGs are only identified in just one study.

### 3.2 Summary of IL-6 related datasets

After screening the GEO database, 13 transcriptome datasets related to IL-6 treatment in *Homo sapien*s were collected for further analyses (Table 1). In these datasets: eight involved direct exogenous treatment with IL-6; three involved treatment using monoclonal antibody inhibitors of IL-6 receptors: one was divided into high and low expression of IL-6 according to measurement in peripheral blood; one involved withdrawal of IL-6 stimulation after treatment for a period of time. The cells used in the experiments included HK-2 cells and dendritic cells. The datasets with the largest number of samples had 18 experimental-group samples and 18 control-group samples. The 13 datasets contained 61 experimental samples and 61 control groups. Because of differences in the genes in each dataset, finally we integrated the 9121 genes detected in all 13 datasets for further analyses.

Using the traditional method to analyze transcriptome datasets, we compared the genes with twofold significant difference. We found that only two genes, *EGR2* and *LYPD1*, had a twofold significant difference in at least two datasets (Table 2). The trend in upregulation and downregulation of *LYPD1* expression in the two datasets was contrary. Only *EGR2* had the same trend in upregulation and downregulation of expression in the two datasets, and had statistical significance. There were >9000 genes, most of which had significant twofold differences in only one dataset (usually GSE12385).

**Table 2:** EGR2 and LYPD1 have two-fold-change in more than one experiment dataset. Note LYPD1 were reversely changed in two datasets. The most significantly changed genes were detected from GSE12385. mean. e indicates mean of experiment samples; mean. c, mean of control samples.

gene	mean.e	sd.e	mean.c	sd.c	p values	Fold	Datasets
*EGR2*	379.06	28.19	105.11	6.26	0.0448558	3.61	GSE68941
*EGR2*	1544.48	1843.89	436.79	590.29	0.0228731	3.54	GSE12385
*LYPD1*	431.56	40.47	189.09	10.40	0.0490625	2.28	GSE68941
*LYPD1*	478.13	92.58	1090.46	24.78	0.0478792	0.44	GSE68942-1
*MARCO*	6527.22	1538.92	3197.50	1411.02	3.39E-07	2.04	GSE12385
*C1QA*	533.28	231.83	211.11	77.38	6.31E-06	2.53	GSE12385
*TMEM51*	1531.78	796.52	541.61	177.03	2.03E-05	2.83	GSE12385
*ERAP2*	8611.11	4251.11	3077.78	2329.74	4.58E-05	2.80	GSE12385
*NRCAM*	27.57	16.21	55.34	18.70	5.689E-05	0.50	GSE12385

### 3.3 IL6 regulated genes identified by meta-analysis

In general, 352 genes were identified using the random-effect models with p < 0.05 to denote significance. Among these 352 genes, expression of 237 genes was downregulated, whereas that of 115 genes was upregulated, after IL6 treatment. Table 3 lists the top-10 genes with the most significant upregulation, among which *ICAM1*, *LDLR* and *SERPINA1* had the most significant upregulation, with a p-value of 3.88 × 10^–4^, 5.65 × 10^–4^ and 8.02 × 10^–4^, respectively. Table 3 lists the top-10 genes with the most significant downregulated expression, among which *DCHS1* had the greatest (1.08 × 10^–3^). Among these genes, the adjusted statistical mean difference was largest for *SOCS3* (1.32) (Table 3).

**Table 3:** The 10 most significantly up- and down-regulated genes in IL-6 treated samples. All parameters were calculated for meta-analysis using Random Effect Model. W, weight; TE, averaged difference; lower and upper, 95% confidence interval; random, random effective model; Heter, the heterogeneity of data.

Gene Symbol	W	TE	lower	upper	Z	random_ pvalue	heter_ pvalue
Top 10 most significantly up-regulated genes							
*ICAM1*	0.35	0.89	0.40	1.38	3.55	3.88E-04	0.32
*LDLR*	0.51	0.70	0.30	1.09	3.45	5.65E-04	0.67
*SERPINA1*	0.97	0.71	0.29	1.12	3.35	8.02E-04	0.43
*SBNO2*	0.32	0.64	0.25	1.04	3.20	1.37E-03	0.50
*MOSPD2*	1.46	0.62	0.24	1.00	3.20	1.39E-03	0.97
*PFKFB3*	0.12	0.76	0.29	1.24	3.15	1.64E-03	0.34
*SOCS3*	0.16	1.32	0.48	2.15	3.10	1.94E-03	0.02
*SUPT3H*	0.09	0.61	0.22	1.00	3.09	1.98E-03	0.81
*TGFA*	0.50	0.70	0.25	1.14	3.05	2.32E-03	0.37
*STOM*	0.93	0.58	0.19	0.98	2.92	3.50E-03	0.46
Top 10 most significantly down-regulated genes							
*DCHS1*	1.37	−0.65	−1.04	−0.26	−3.27	1.08E-03	0.72
*SLC24A1*	1.09	−0.64	−1.03	−0.25	−3.21	1.35E-03	0.51
*NDRG3*	1.49	−0.62	−1.01	−0.24	−3.17	1.53E-03	0.94
*TPCN1*	0.13	−0.61	−1.00	−0.23	−3.12	1.83E-03	0.50
*OSGEPL1*	0.85	−0.59	−0.97	−0.21	−3.05	2.29E-03	0.94
*TOMM20*	1.50	−0.57	−0.94	−0.19	−2.97	2.98E-03	0.92
*PREPL*	0.67	−0.59	−0.97	−0.20	−2.97	3.00E-03	0.85
*LIPA*	0.45	−0.58	−0.96	−0.19	−2.94	3.31E-03	0.71
*SLC25A40*	1.29	−0.58	−0.96	−0.19	−2.93	3.36E-03	0.69
*ISYNA1*	0.40	−0.58	−0.96	−0.19	−2.92	3.50E-03	0.64

We also checked the overlapping between our results and COVID-19 clinical studies. As shown in Fig. 1B, 120 DEGs were identified by both our meta-analysis and other COVID-19 clinical studies, and 232 DEGs were only found by our method. A co-expression network was built to show interaction in 352 genes identified from our studies. Fig. 1C displayed that several co-expressions are exist among these genes.

### 3.4 Enrichment analyses using the GO database revealed the biological function of IL-6-regulated genes

To understand the biological functions of DEGs, we utilized enrichment analyses using the GO database to classify genes into three groups: biological process, cell compartment and molecular function.

**Fig. 2:**
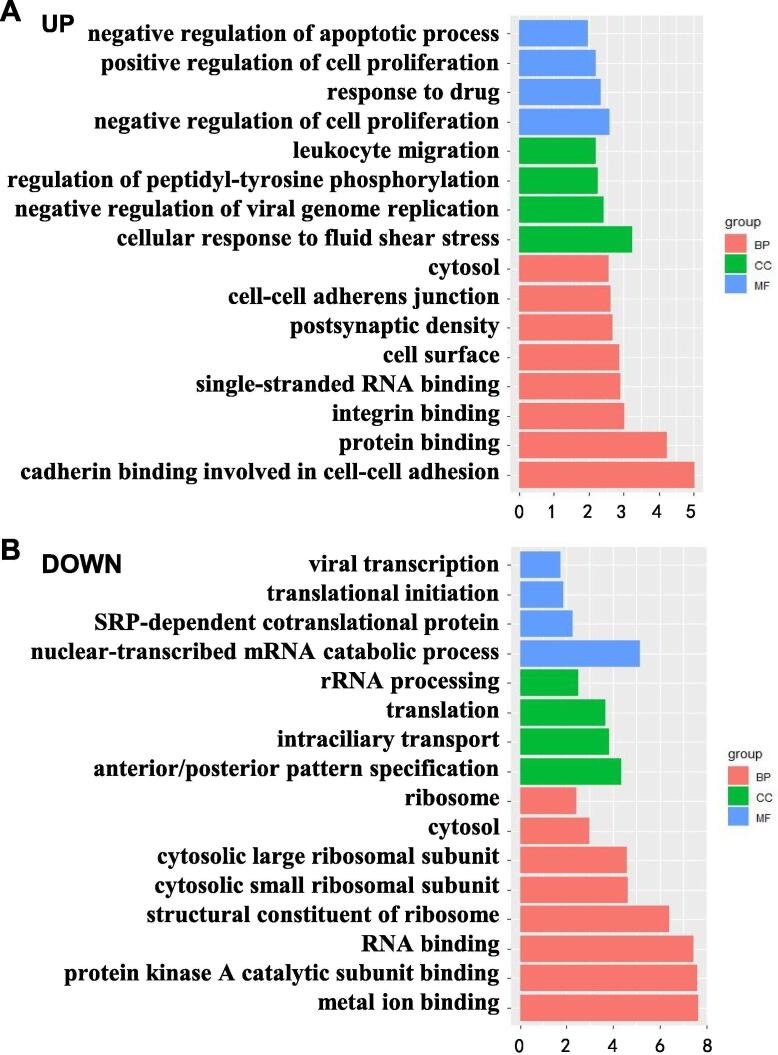
Enrichment analyses (using the GO database) of differentially expressed genes after IL-6 treatment. (A) The most significantly enriched GO terms for upregulated genes. (B) GO terms for downregulated genes. BP, biological process; CC, cell compartment; MF, molecular function.

The biological processes of the upregulated 115 genes were enriched mainly in: negative regulation of apoptotic process (9.61 × 10^–6^), response to drug (9.26 × 10^–4^), leukocyte migration (1.29 × 10^–3^) and negative regulation of virtual genome replication (2.43 × 10^–3^). The main cell compartments were located in the cytosol (5.63 × 10^–4^), and cell adherence (3.82 × 10^–3^) was also important. The main molecular function was single-stranded RNA binding (2.54 × 10^–3^) (Fig. 2A).

The 237 downregulated genes were enriched in the biological processes of virtual transcription (2.27 × 10^–8^), translation initiation (2.55 × 10^–8^), SRP-dependent co-translational protein targeting to the membrane (3.52 × 10^–8^), and nuclear transcribed mRNA catabolic process, nonsense mediated calcium (4.05 × 10^–7^). The molecular function was the structural condition of ribosomes (7.14 × 10^–6^) (Fig. 2B).

### 3.5 Analyses of the KEGG database suggested IL-6-regulated genes to be involved in typical pathways

**Fig. 3:**
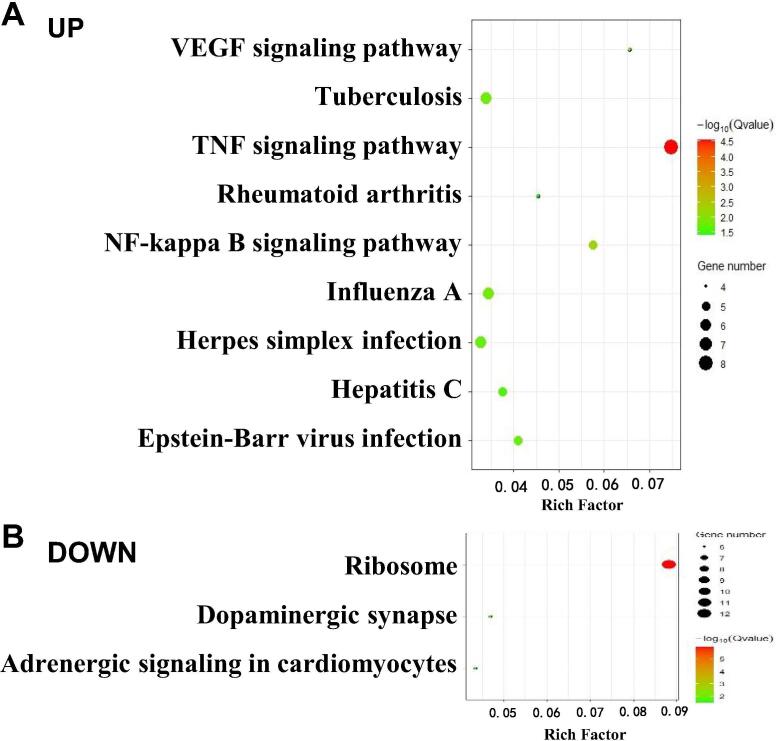
Enrichment analyses (using the KEGG database) of differentially expressed genes after IL-6 treatment. Upregulated genes (A) and downregulated genes (B) were enriched in different pathways.

Analyses of pathway enrichment were done using the KEGG database for IL-6-regulated genes. The biological processes of the upregulated 115 genes were enriched mainly in the TNF signaling pathway (2.83 × 10^–5^), NF-κB signaling pathway (6.02 × 10^–3^), influenza A (1.52 × 10^–2^) and rheumatoid arthritis (3.77 × 10^–2^) (Fig. 3A). The 237 downregulated genes were enriched in the ribosome (1.18 × 10^–6^), dopaminergic synapse (2.47 × 10^–2^) and adrenergic signaling in cardiomyocytes (3.28 × 10^–2^) (Fig. 3B).

### 3.6 IL-6-regulated genes were associated with several diseases

**Fig. 4:**
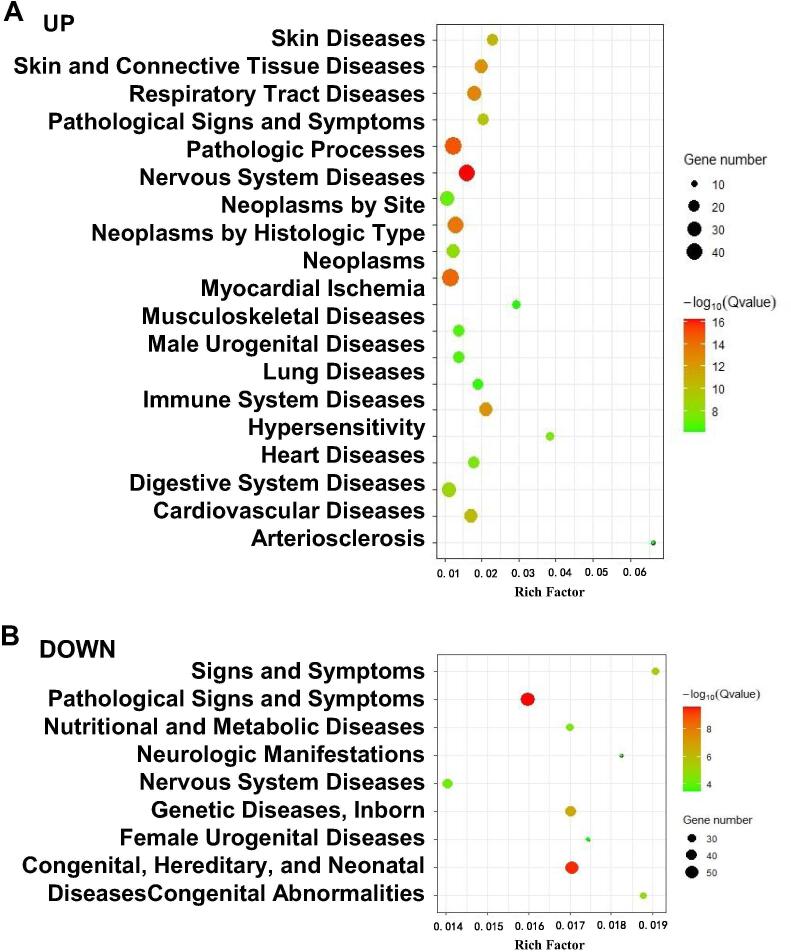
Enrichment analyses of the disease-associated genes regulated by IL-6. Upregulated genes (A) were enriched in respiratory-tract diseases and cardiovascular diseases. Downregulated genes (B) were enriched in pathological signs and symptoms.

Enrichment analyses were also carried out for associated diseases according to the CTD [27]. Fig. 4A displays the top-20 most significantly enriched diseases for upregulated genes, including pathological processes (8.50 × 10^–20^), neoplasms (6.53 × 10^–18^), skin and connective tissue diseases (1.11 × 10^–16^), immune-system diseases (4.35 × 10^–16^), cardiovascular diseases (4.98 × 10^–14^), respiratory diseases (2.71 × 10^–13^), male urogenital diseases (3.45 × 10^–10^) and lung diseases (7.55 × 10^–10^).

Fig. 4B shows the top-9 the most significantly enriched diseases for downregulated genes, including pathological conditions, signs and symptoms (2.64 × 10^–10^), congenital, hereditary, and neonatal diseases and abnormalities (4.06 × 10^–10^) and nervous-system diseases (6.88 × 10^–5^).

### 3.7 DEGs with fold-change

**Fig. 5:**
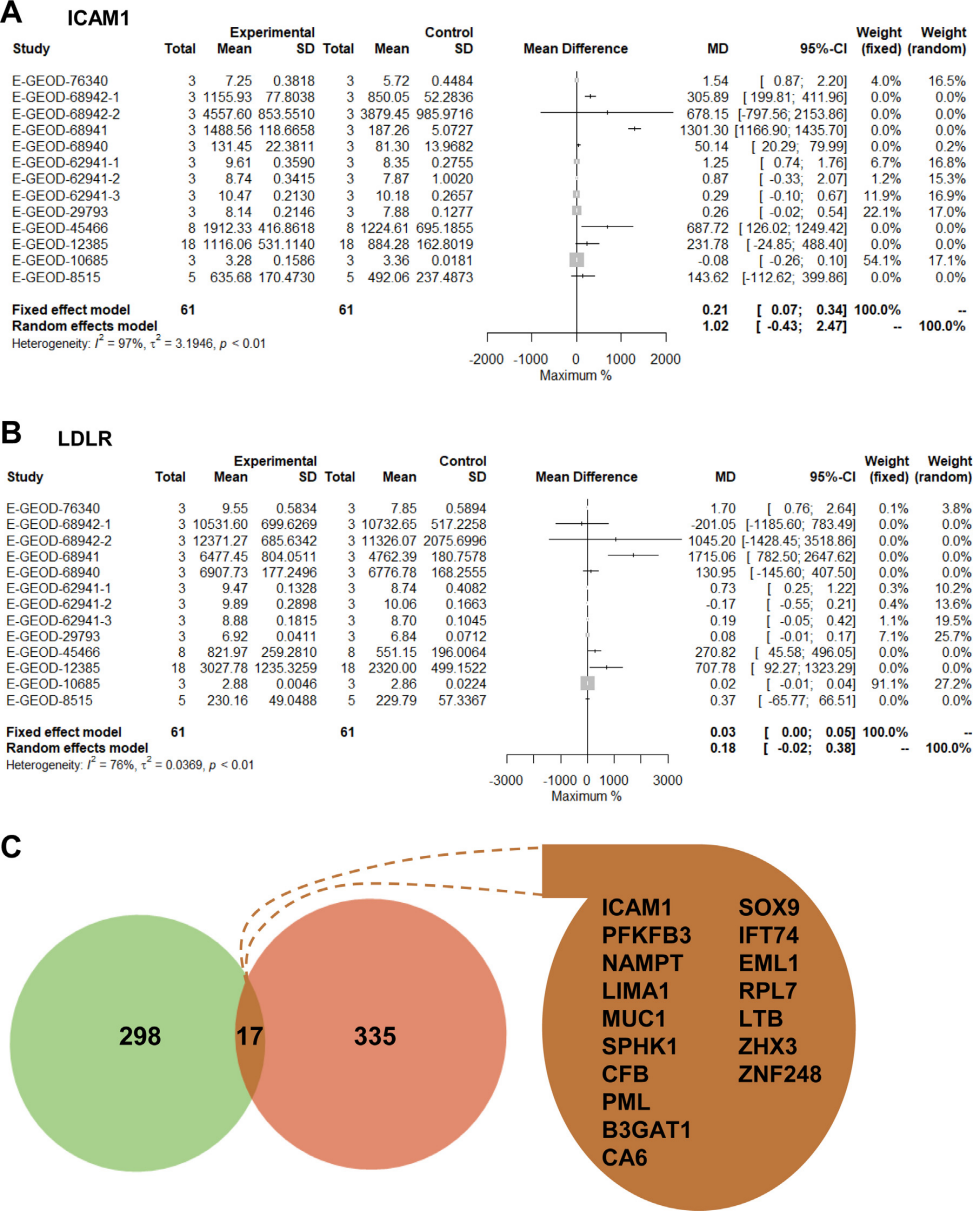
Analyses of fold-change in differentially expressed genes identified by the meta-analysis. Random forest map for ICAM1 (A) and LPLR (B) in the meta-analysis. (C) Traditional analyses of fold-change in a transcriptome dataset shows overlap with genes discovered in the meta-analysis. Green, traditional analyses of fold-change; red, meta-analysis. (For interpretation of the references to colour in this figure legend, the reader is referred to the web version of this article.)

According to the results of the meta-analysis of *ICAM1* and *LDLR* (the two genes with the most significant changes in expression), using the random-effects model, the combined average difference (95% confidence interval (CI) was 1.02 (0.43–2.47) and 0.18 (0.02–0.38), respectively. However, in E-GEOD-68941 alone, the mean fold-change of *ICAM1* in the experimental group was more than twofold compared with that in the control group, but *LDLR* did not have a greater-than-twofold change in any dataset (Fig. 5A, B). In total, 315 genes had a greater-than-twofold change in at least one dataset (Fig. 4C). Only 17 genes overlapped with the DEGs in the meta-analysis, including *ICAM1*, *PFKFB3*, *NAMPT* and *LIMA1* (Fig. 5C).

### 3.8 *ICAM1* and *PFKFB3* expression was induced in patients with severe COVID-19

**Fig. 6:**
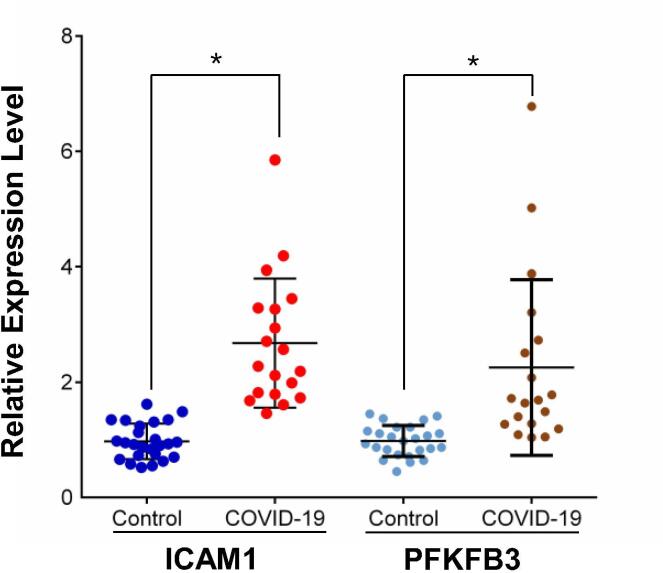
Increased expression of *ICAM1* and *PFKFB3* in patients with severe COVID-19. Relative expression of *ICAM1* and *PFKFB3* determined by qRT-PCR in patients with severe COVID-19 and in healthy controls. *p < 0.001.

RNA expression of *ICAM1* and *PFKFB3* in the blood plasma of patients with severe COVID-19 and healthy controls was measured by qRT-PCR. Expression of ICAM1 and PFKFB3 was significantly induced in severe-COVID-19 patients compared with that in healthy controls (Fig. 6).

## 4 Chemical–gene interactions interfere with IL-6 regulation

CTD Database could predict the interaction between gene and chemicals which are potential drug for disease. The evidence of interaction between chemicals and genes, such as direct protein binding or inhibition of protein activity, was collected from the CTD. Fourteen genes and 111 chemicals had an interaction. Several chemicals could interact with multiple genes. Copper interacted with five genes: *ICAM1*, *LIMA1*, *CFB*, *SOX9* and *ZHX3* (Table 4). Resveratrol interacted with *NAMPT*, *SPHK1*, *PML* and *SOX9* (Table 4).

**Table 4:** Chemical-gene interactions.

Chemical Name	Chemical ID	Gene Symbol	Interaction Actions	PubMedIDs
Copper	D003300	ICAM1	decreases:reaction|increases:expression	12633744
Copper	D003300	LIMA1	affects:binding|increases:expression	20971185
Copper	D003300	CFB	affects:binding	23896426
Copper	D003300	SOX9	affects:binding|decreases:expression	20971185
Copper	D003300	ZHX3	affects:binding|increases:expression	24690739
Resveratrol	D000077185	NAMPT	decreases:reaction|increases:activity	24603648
Resveratrol	D000077185	SPHK1	affects:localization|decreases:activity	26045781
Resveratrol	D000077185	PML	affects:binding|decreases:reaction|increases:reaction	19631782
Resveratrol	D000077185	SOX9	affects:binding|affects:response to substance	24962570
Enzyme Inhibitors	D004791	LIMA1	decreases:activity|increases:O-linked glycosylation	23301498
Enzyme Inhibitors	D004791	PML	decreases:activity|increases:O-linked glycosylation	23301498
Enzyme Inhibitors	D004791	RPL7	decreases:activity|increases:O-linked glycosylation	23301498

## 5 Discussion

Aim of this study is to understand the molecular and cellular processes involved in severe COVID-19. Considering the importance of IL-6 in COVID-19, it is rational to identify the genes and pathways regulated by IL-6. Multiple publicly available transcriptome datasets underwent meta-analysis for the transcriptome characteristics of IL-6 treatment. Using this strategy, we identified the DEGs in different types of samples, and described their biological functions, pathways (using GO and KEGG databases) and the diseases they were associated with.

Collection of transcriptome information under IL-6 treatment revealed that virtually no gene had repeatable twofold changes in expression. This finding may have been because different datasets had different treatment methods for IL-6, or because use of different cell types/tissues led to different gene responses. Nevertheless, DEGs with a significant difference in expression were identified by our meta-analysis. The same response was found for genes from different cell types and under different treatment conditions, suggesting that these genes were likely to be key genes for IL-6 regulation in various conditions.

Meta-analysis re-evaluated the statistics by considering the sample size of each experiment. Based on this, we found that DEG has more statistical repeatability in different conditions and a large number of cases. Compared with the published clinical transcriptional profiling studies of COVID-19, these studies used only a few cases, and the samples were different tissues. In the results of these studies, the number of repeated DEG was relatively small (Fig. 1). This suggests that our method has potential advantage than clinical research to save time and increase the accuracy.

Our meta-analysis disclosed some significant differences in gene expression after IL-6 treatment (Table 3). Many of the genes we found to be upregulated by IL-6 are supported by experimental evidence. Among them, *ICAM1* (a well-known gene involved in viral infection and cardiovascular disease) expression has been demonstrated to be consistent with IL-6 expression in mouse macrophages [28], [29]. Some treatments, such as Phoenix 20 and soluble matrine 2, can induce the co-expression of ICAM1 and IL-6 [30], [31]. In human astrocytes, IL-6 can upregulate *SBNO2* expression [32]. IL-6 can induce *PFKFB3* expression [33] through the STAT3 signaling pathway. *TGFA* expression has a positive correlation with IL-6 expression [32]. However, expression of some genes showed the opposite trend in other experiments. In some studies, IL-6 expression was found to be negatively correlated with SERPINA1 expression [34]. In other studies, expression of IL-6 and SERPINA1 was increased with disease progression [35], [36]. SOCS3 expression has been shown to be negatively correlated with IL-6 expression [37], [38], [39], [40]. A negative correlation between expression of TPCN1 and TOMM20 and IL-6 expression has been documented [40], [41]. However, contrary to our findings, studies showed that expression of IL-6 and LIPA decreased in the treatment of some diseases [42]. High expression of IL-6 has been noted in critically ill patients with COVID-19, and IL-6 expression could be used to assess risk. Our meta-analysis showed that expression of *ICAM1* and *PFKFB3* was increased in COVID-19 patients. These genes could be used as biomarkers in COVID-19.

We found expression of *ICAM1* and *PFKFB3* to be enriched in virus-related regulatory pathways, the NF-κB signaling pathway, and apoptosis-related pathways (Fig. 2, Fig. 3). As an important cytokine in the immune system, IL-6 has been shown to be closely related to the pathways mentioned above in several studies. Through analyses of the KEGG database, we found expression of PRKCA, SCN1B, PLCB4, CAMK2G, CREB3L1 and PPP1CC (which are negatively regulated by IL-6) to be enriched in adrenergic signaling in cardiomyocytes. Only a few studies have found that IL-6 regulates the response to adrenaline stimulation through the mitogen-activated protein kinase (MAPK) pathway in mouse cardiomyocytes [43]. *ICAM1* is a critical player in heart diseases. Evidence suggests that inhibition of ICAM1 expression could be novel treatment for hypertrophic heart diseases [44]. In the current outbreak of COVID-19: (i) the CRS caused by IL-6 has been an important cause of death; (ii) patients with heart disease or diabetes mellitus have a high prevalence of death. PFKFB3 has been suggested to have a critical role in diabetes mellitus [45]. We identified PFKFB3 to be enriched in the present study.

We conducted a more extensive study (through the CTD) on the relationship between disease and the genes found to be regulated by IL-6 in our meta-analysis. The IL-6 regulatory genes were closely associated with vascular diseases, cardiovascular diseases, respiratory diseases, and diabetes mellitus. Digestive-system releases, hypersensitivity, male urogenital releases, and nervous-system diseases were also enriched by genes regulated by IL-6, so we could consider a combination of these diseases in the CRS elicited by IL-6. Finally, we collected drug information related to IL-6 regulatory genes from the CTD database and found that resveratrol interacted with four key genes. This finding could aid development of treatment to counteract CRS during COVID-19.

## Declaration of Competing Interest

The authors declare that they have no known competing financial interests or personal relationships that could have appeared to influence the work reported in this paper.
